# Non-pharmacological interventions on anxiety and depression in lung cancer patients’ informal caregivers: A systematic review and meta-analysis

**DOI:** 10.1371/journal.pone.0282887

**Published:** 2023-03-13

**Authors:** Fang Lei, Eunice Lee, Joosun Shin, Shin-Young Lee

**Affiliations:** 1 School of Nursing, University of Minnesota, Twin Cities, MN, United States of America; 2 School of Nursing, University of California, Los Angeles, Los Angeles, CA, United States of America; 3 School of Nursing, University of California, San Francisco, San Francisco, CA, United States of America; 4 Department of Nursing, Chosun University, Gwangju, Republic of Korea; University of Technology Sydney, AUSTRALIA

## Abstract

**Background:**

Lung cancer is one of the common cancers and the leading cause of death. Tremendous caregiving burden of informal caregivers of lung cancer causes psychological disorders, such as anxiety and depression. Interventions for informal caregivers of patients with lung cancer to improve their psychological health, which ultimately leads to patients’ positive health outcomes, are crucial. A systematic review and meta-analysis was conducted to: 1) evaluate the effect of non-pharmacological interventions on the outcomes of depression and anxiety for lung cancer patients’ informal caregivers; and 2) compare the effects of interventions with differing characteristics (i.e. intervention types, mode of contact, and group versus individual delivery).

**Methods:**

Four databases were searched to identify relevant studies. Inclusion criteria for the articles were peer-reviewed non-pharmacological intervention studies on depression and anxiety in lung cancer patients’ informal caregivers published between January 2010 and April 2022. Systematic review procedures were followed. Data analysis of related studies was conducted using the Review Manager Version 5.4 software. Intervention effect sizes and studies’ heterogeneity were calculated.

**Results:**

Eight studies from our search were eligible for inclusion. Regarding total effect for the caregivers’ levels of anxiety and depression, results revealed evidence for significant moderate effects of intervention on anxiety (SMD -0.44; 95% CI, -0.67, -0.21; p = 0.0002) and depression (SMD -0.46; 95% CI, -0.74, -0.18; p = 0.001). Subgroup analyses for both anxiety and depression of informal caregivers revealed moderate to high significant effects for specific intervention types (cognitive behavioral and mindfulness combined with psycho-education interventions), mode of contact (telephone-based interventions), and group versus individual delivery.

**Conclusion:**

This review provides evidence that cognitive behavioral and mindfulness-based, telephone-based, individual or group-based interventions were effective for informal caregivers of lung cancer patients. Further research is needed to develop the most effective intervention contents and delivery methods across informal caregivers with larger sample size in randomized controlled trials.

## Introduction

Lung cancer is the second most common cancer in both men and women and the leading cause of cancer death among both men and women, making up almost 21% of all cancer deaths [1]. National Cancer Institute [1] estimates that about 236,740 new lung cancer cases will occur, and about 130,180 patients with lung cancer will die in 2022.

Treatments and care for patients with lung cancer have been advanced gradually, but many unsolved issues remain. Lung cancer patients experience significant physical and psychosocial symptoms, including pain, dyspnea, anorexia, anxiety, and depression due to cancer itself and/or its treatment. Compared to other types of cancer, lung cancer patients had a higher symptom burden, resulting in poor quality of life [2, 3]. Because of the disease trajectory of lung cancer patients, informal caregivers play a key role in caring for them [4]. Informal caregivers can assist patients with lung cancer in managing symptoms, activities of daily living, finance, transportation, seeking information, and providing psychosocial support [4–7]. Taking care of cancer patients is a tremendous burden for informal caregivers [8] and the resultant burden often leads to in physical as well as psychosocial malfunction [4, 9]. In particular, psychological disorders such as anxiety and depression are prevalent and frequently occur in patients with lung cancer and their informal caregivers [4, 9]. For example, studies [5, 9] have reported that psychological symptoms including anxiety (32.6%-37%) and depression (22%-25.5%) are prevalent among the caregivers of cancer patients. Higher levels of depression and anxiety for caregivers are associated with impaired quality of life of cancer patients, younger patients, or patients’ physical function declined (p<0.05) [10, 11]. Family caregivers of cancer patients are distressed by the poor quality of their own life, disruption of their usual social activities, and the emotional and physical burden of caregiving (p<0.05) [12]. Caregivers of cancer patients desperately need interventions to address their psychosocial problems. One study [6] provided evidence that 36% of caregivers of cancer patients (N = 99) reported the most difficult part of caregiving was psychosocial; 31% of caregivers responded that they need more information to help them cope emotionally.

Maintaining the psychological health of informal caregivers is all the more important because it is associated with the clinical health of cancer patients [7, 13, 14]. For example, a study of 43 lung cancer patient and caregiver dyads found that patient’s symptoms were positively correlated with the caregiver’s depression and anxiety [7]. Another study [15] linked higher depression scores of the patient with caregiver depression (b = 0.72, p<0.001). For these reasons, the Clinical Practice Guidelines for Quality Palliative Care published by the National Coalition for Hospice and Palliative Care [16] suggest that palliative care should focus on not only physical, psychological, functional, spiritual, and practical aspects of seriously ill patients but should also be family-centered, emphasizing the importance of family caregiver assessment, support, and education.

In the past, many systematic reviews and meta-analyses [17–19] have explored the effectiveness of interventions on anxiety and depression for lung cancer patients. A number of systematic reviews and meta-analyses have examined the effects of non-pharmacological interventions on anxiety and depression for informal caregivers of people with cancer. These meta-analyses have found inconsistent results between and within different kinds of interventions. For example, a meta-analysis of meditation intervention [20] has shown statistically significant improvement in depression and anxiety in informal caregivers, while another of cognitive behavioral therapies [21] and psychosocial interventions [22] were less favorable. A Cochrane Systematic review and meta-analysis [23] found that psychosocial interventions have a significant effect on depression but insignificant effect on anxiety in caregivers of advance cancer patients. Up to date, inconsistent results of meta-analyses on non-pharmacological interventions on anxiety and depression in informal caregivers of cancer patients have been reported. Furthermore, none have examined non-pharmacological interventions on anxiety and depression for the informal caregivers of lung cancer patients. Investigating the topic further will fill the gap in the literature, also findings from this study could help to inform effective intervention strategies to mitigate the prevalent psychological problems (e.g., anxiety and depression) in informal caregivers of lung cancer patients, which will eventually help to decrease their psychological burden. This systematic review and meta-analysis is the first to appraise the effectiveness of non-pharmacological interventions to reduce anxiety and depression in informal caregivers of lung cancer patients. More specifically, a systematic review and meta-analysis was conducted to: 1) evaluate the effect of non-pharmacological interventions on the outcomes of depression and anxiety for lung cancer patients’ informal caregivers; and 2) compare the effects of interventions with differing characteristics (i.e. intervention types, mode of contact, and group versus individual delivery).

## Methods

We followed the Preferred Reported Items for Systematic Reviews and Meta-Analyses (PRISMA) guidelines.

### Search strategy

We used the following electronic databases (EMBASE, CINHAL, PsycInfo, and PubMed) and Clinical Trials.gov. The database search strategy used a combination of medical subject headings (MeSH) terms and text keywords. An example of our PubMed search strategy is in the appendix. The main keywords were informal caregivers, patients with lung cancer, and intervention. Detailed keywords for the literature search were: 1) informal caregivers: caregivers, family members, relatives, or carers; 2) patients with lung cancer: lung cancer, lung neoplasms, lung tumor or lung adenocarcinoma; 3) intervention: intervention, program, education, training, patient education, patient teaching, psychotherapy, support education, or communication; 4) depression: depression, depressive disorder, depressive symptoms, and major depressive disorder; and 5) anxiety: anxiety, anxiety disorders, and generalized anxiety disorder. We also hand-searched reference lists of full-text manuscripts and cross-referenced for potentially relevant papers.

### Eligibility criteria and study selection

We included primary intervention articles published between January 2010 and April 2022 in English. Additional inclusion criteria for the articles included: 1) population: targeted population is lung cancer patients’ informal caregivers (defined as family members, friends, or anyone who assisted the patients with lung cancer without compensation); 2) intervention: peer-reviewed studies on non-pharmacological interventions (defined as health interventions that were not primarily based on medication), and 3) outcome: outcomes included depression and/or anxiety. We excluded articles that 1) included various types of cancer (including lung cancer) but did not report data pertinent to the subgroup of informal caregivers; 2) reported only intervention development processes without results; and 3) informal articles such as conference abstracts or commentary articles. The first and the second authors reviewed all abstracts from the search results and selected studies for full text review. Two authors of this study independently screened the titles and abstracts of relevant studies.

### Data extraction

Data about the study characteristics (first author and year, country, study design, sample size), intervention type (cognitive-based, mindfulness, yoga, or meditation), mode of contact (in person or telephone-based), group versus individual delivery, and anxiety and depression outcomes were extracted from each study. The first author extracted the data and the second author verified it. No disagreement existed between the two authors regarding the data extraction results.

### Quality assessment

We used the Physiotherapy Evidence Database (PEDro) scale [24] to determine the quality of the intervention articles. The PEDro scale is used for randomized clinical trial (RCT) studies to determine internal validity [24]. The scale has 11 items, the higher the score, the greater the methodological quality. We included non-RCT studies due to the limited available intervention articles, but the PEDro scale was still useful in evaluating the quality of articles.

### Data analysis

We used the Review Manager Version 5.4 software to conduct the meta-analysis. A random-effects model was applied in the analysis since the observed estimates of intervention effect varied across studies both due to real differences in the intervention effect in each study and sampling variability [25]. Exploratory post-hoc subgroup analyses were conducted to examine the effects of the intervention types, mode of contact, and group versus individual delivery on the anxiety and depression of lung cancer patients’ informal caregivers. Intervention effect sizes for anxiety and depression symptoms were calculated using Hedge’s g statistic and weighted by the sample size of the studies. The Hedge’s g-values were then averaged to calculate the overall effect size and converted to a z value. Hedge’s g was interpreted as 0.2 to indicate a small, 0.5 a medium, and 0.8 a large effect size [26]. The Tau^2^ and I^2^ statistics were utilized to evaluate the included studies’ heterogeneity and reveal the variance among the studies. The I^2^ statistics values were categorized into no (0%–25%), low (25%–50%), moderate (50%–75%), and high (75%–100%) heterogeneity [27]. Since the studies used different measurement scales to measure informal caregivers’ anxiety and depression levels, we used the standardized mean differences along with its 95% confidence intervals to measure the estimated effect size. Because some of studies were quasi-experimental, without control groups, data from control groups in the randomized control trial studies and pre-intervention data from quasi-experimental studies were used as comparisons in evaluating the effect of interventions. We assessed the risk of publication bias within studies according to PRISMA recommendations. Moreover, forest plots were prepared to visualize the effect size and the standardized mean difference with 95% CI. Publication bias was examined visually using funnel plots. An asymmetrical funnel plot represents a potential publication bias. The first author did the data analysis, and the second author reviewed and verified the results.

## Results

### Study selection

**Fig 1:**
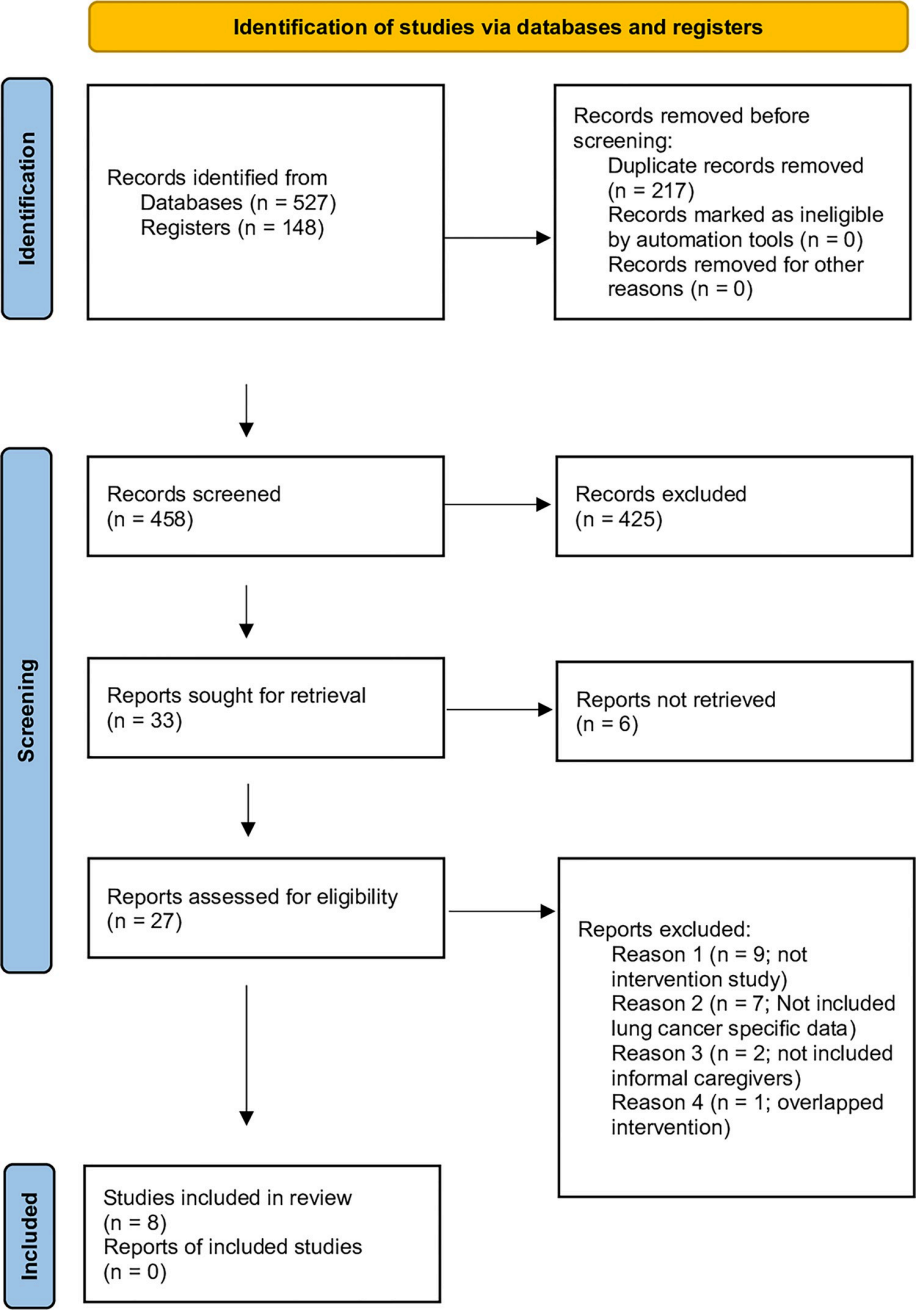
PRISMA flow chart of the systematic review process.

We identified 594 articles, of which 8 were included [28–35]. See Fig 1 for a flow chart of the systemic review process using the PRISMA.

### Study characteristics

Characteristics of the studies are summarized in Tables 1 and 2. All of the 8 studies were published between 2015 to 2020 [28–35]. Five of them were conducted in the United States [28, 30–33]. Of the remaining three, one study was conducted in China [29] and the other two were conducted in Netherlands [34] and Cyprus [35]. The sample size of included studies ranged from 9 [33] to 120 [29]. Four studies were randomized controlled trials [28–31] and the remaining four studies used quasi-experimental design [32–35].

**Table 1:** Study characteristics of the included studies.

Citation (first author, date)	Country	Study design	Instrument	Sample Size of Caregivers		Caregivers Outcome results Mean/Median (standard deviation)	
				Intervention group	Comparison group	After intervention/intervention group	Before intervention/comparison group
1. Badr et al. (2015)	United States	Two group randomization feasibility study	6-item PROMIS short-form	20	19 (usual care)	Anxiety 12.1 (3.60) Depression 11.5 (3.20)	Anxiety 17.16 (5.41) Depression 16.53 (5.47)
2. Choratas et al. (2020)	Cyprus	Two group randomization feasibility study	Hospital Anxiety and Depression Scale	11	8 (usual care)	Anxiety 9.5 (3.5) Depression 7.9 (4.3)	Anxiety 8.8 (3.1) Depression 8.6 (4.2)
3. Li et al. (2018)	China	Prospective intervention pilot study, randomized control study	Hospital Anxiety and Depression Scale	58	62 (usual care)	Anxiety -3.1 (5.6) Depression -2.9 (2.4)	Anxiety -0.9 (2.4) Depression -0.5 (3.9)
4. Milbury, Chaoul et al. (2015)	United States	One group pilot study	Brief Symptom Inventory-18	10	NA	Anxiety 0.42 (0.44) Depression 10.55 (9.45)	Anxiety 0.76 (0.60) Depression 13.11 (6.91)
5. Milbury, Mallaiah et al. (2015)	United States	One group feasibility study	Centers for Epidemiological Studies-Depression; Brief Symptom Inventory-18	9	NA	Anxiety 0.46 (0.52) Depression 0.38 (0.28)	Anxiety 0.44 (0.50) Depression 0.29 (0.39)
6. Mosher et al. (2016)	United States	Two groups, randomized pilot study	Patient Health Questionnaire-8; Generalized Anxiety Disorder seven-item scale	51	55 (education/support condition)	Anxiety 5.06 (4.28) Depression 5.09 (4.88)	Anxiety 6.51 (6.04) Depression 5.89 (5.22)
7. Mosher et al. (2019)	United States	Pilot feasibility randomized control study	4-item PROMIS	25	25 (education/support condition)	Anxiety 7.23 (0.69) Depression 5.80 (0.62)	Anxiety 7.68 (0.72) Depression 6.20 (0.65)
8. van den Hurk et al. (2015)	Netherlands	One group pilot mixed methods study	Hospital Anxiety and Depression Scale	11	NA	Anxiety 9.4 (4.0) Depression 6.3 (3.6)	Anxiety 10.6 (6.8) Depression 8.1 (3.9)

**Table 2:** Characteristics of the interventions in the included studies.

Citation (first author, date)	Intervention types	Mode of contact	Group versus individual delivery	Intervention frequency	Intervention duration	Intervention components
1. Badr et al. (2015)	Cognitive-behavioral interventions (CBI)	Telephone-based	Individual-based	weekly	60 mins per week, 6 weeks totally	Psychosocial intervention (self-care, stress and coping, symptom management, effective communication, problem solving, and maintaining and enhancing relationships)
2. Choratas et al. (2020)	Breathlessness education and practice	In person	Group-based	Once every two weeks	30–50 mins per session, 2 sessions totally	A PowerPoint presentation incorporating two video recordings and a practical exercise
3. Li et al. (2018)	CBI	In person	Group-based	3 sessions each week	30 mins each session, participants chose to attend at least 4 sessions	Wellness education (information about treatment, diet, social needs, rehabilitation, physical/mental health education, communication strategies, patient care advice)
4. Milbury, Chaoul et al. (2015)	Yoga/meditation	In person	Individual-based	weekly	45–60 mins per week, 2–3 weeks	The program combining yoga techniques and meditation.
5. Milbury, Mallaiah et al. (2015)	Yoga/meditation	In person	Individual-based	weekly	60 mins per week, 2–3 weeks	The program combining yoga techniques and meditation.
6. Mosher et al. (2016)	CBI	Telephone-based	Individual-based	weekly	45 mins per week, 4 weeks totally	Telephone symptom management intervention (relaxation, cognitive restructuring, problem solving, self-soothing, pleasant activities, activity pacing, communication plan for continued skills practice)
7. Mosher et al. (2019)	Mindfulness/psychoeducation	Telephone-based	Individual-based	weekly	50 mins each session, 6 weeks totally	Acceptance and Commitment Therapy includes increasing the skills of mindfulness, perspective-taking, cognitive defusion, acceptance, value clarification, and committed action.
8. Van den Hurk et al. (2015)	Mindfulness/psychoeducation	In person	Group-based	Not mentioned	2.5h each session, 8 sessions totally	Mindfulness-Based Stress Reduction training combining mindfulness practices and psychoeducation.

### Intervention characteristics

Of the eight studies, four studies used cognitive-behavioral interventions or body-mind interventions [28, 29, 31, 35], two studies used mediation/mindfulness [30, 34], two studies used yoga [32, 33]. Regarding the ways in which interventions were delivered, five studies were in person interventions [29, 32–35], while three studies were telephone-based [28, 30, 31]. All the studies used couple-based interventions focusing on both patients with lung cancer and their informal caregivers. Three of the eight studies used group-based interventions [29, 34, 35], and five studies used individual-based interventions [28, 30–33]. All interventions were delivered on a regular basis and were given multiple times, for instance, weekly 60 minute telephone counseling over 6 weeks [28], or four 30 minute sessions over 8 weeks [29]. All interventions had multiple components and were led by health care professionals, such as intervention manuals, telephone counseling sessions [28], and multidisciplinary educational sessions [29]. Intervention effects on anxiety and depression are reported separately.

### Quality evaluation

Overall, the quality of the studies was satisfactory based on the PEDro scale (Table 3). Most of the studies were randomized controlled trials, except for three studies that did not have control groups [32–34]. Two studies had an attrition rate of more than one-third [34, 35]. Therefore, those studies are of relatively lower quality than others.

**Table 3:** Results of quality of study evaluation.

	Badr et al. (2015)	Choratas et al. (2020)	Li et al. (2018)	Milbury, Chaoul et al. (2015)	Milbury, Mallaiah et al. (2015)	Mosher et al. (2016)	Mosher et al. (2019)	van den Hurk et al. (2015)
1. Eligibility criteria were specified.	Y	Y	Y	Y	Y	Y	Y	Y
2. Subjects were randomly allocated to groups	Y	Y	Y	N (No control group)	N (No control group)	Y	Y	N (No control group)
3. Allocation was concealed.	Y	Y	Y	N/A	N/A	Y	Y	N/A
4. The groups were similar at baseline for the most important prognostic Indicators.	Y	Y	Not mentioned	N/A	N/A	Y	Y	N/A
5. All subjects were blinded.	Unclear	N	N	N/A	N/A	Unclear	Unclear	N/A
6. All therapists who administered the therapy were blinded.	N	N	N	N/A	N/A	N	Unclear	N/A
7. All assessors who measured at least one key outcome were blinded.	Y	Not mentioned	Not mentioned	N/A	N/A	Y	Y	N/A
8. Measures of at least one key outcome were obtained from more than 85% of the subjects initially allocated to groups.	Y	N 9/24 (37.5%) dropped	Y	N 3/14 (21.4%) dropped	N 3/13 (23.1%) dropped	Y	Y	N 4/9 (44.4%) dropped
9. All subjects for whom outcome measures were available received the treatment or control condition as allocated or, where this was not the case, data for at least one key outcome was analyzed by “intention to treat”.	Y	Y	Not mentioned	N	N	Y	Y	N
10. The results of between-group statistical comparisons are reported for at least one key outcome.	Y	Y	Y	N/A	N/A	Y	Y	N/A
11. The study provides both point measures and measures of variability for at least one key outcome.	Y	Y	Y	Y	Y	Y	Y	Y

### Effect on the anxiety of informal caregivers for lung cancer patients

#### Total effect for the caregivers’ levels of anxiety

**Fig 2:**
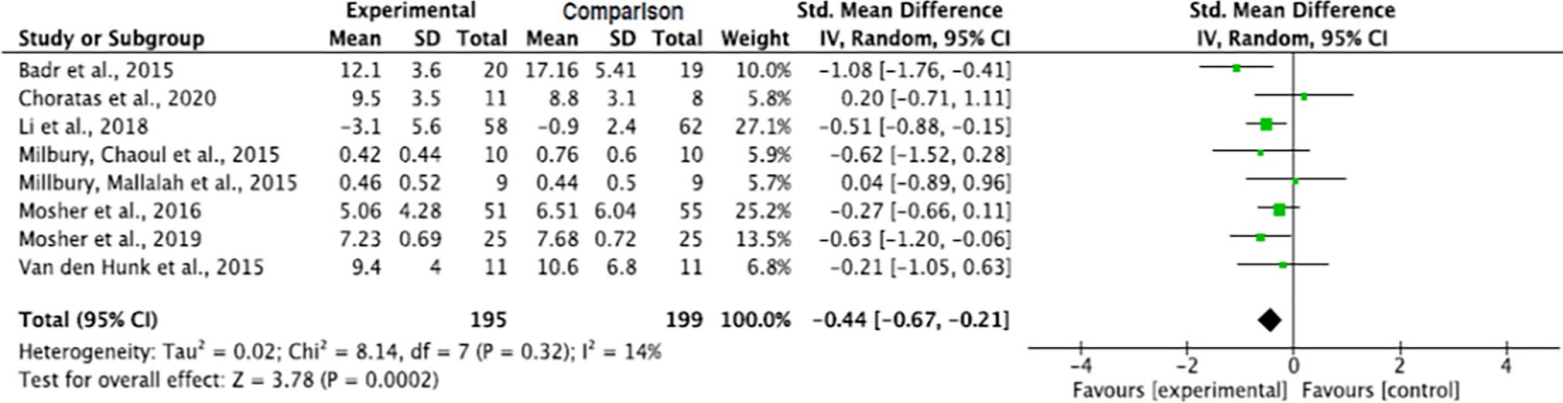
Interventions’ effect on the anxiety of informal caregivers for lung cancer patients.

Overall, interventions on informal caregivers of patients with lung cancer significantly decreased the caregivers’ anxiety levels in the intervention groups, as opposed to the comparison groups (p = 0.0002). The pooled summary effect of the included interventions showed that, post intervention, informal caregivers in the intervention group were 0.44 lower at risk for anxiety as contrasted with the comparison group (SMD, -0.44; 95% CI, -0.67, -0.21). However, a very small heterogeneity was noticed across the study results (Tau^2^ = 0.02, ChI^2^ = 8.14, df = 7, p = 0.32, I^2^ = 14%) (Fig 2).

### Effect by intervention types

**Fig 3:**
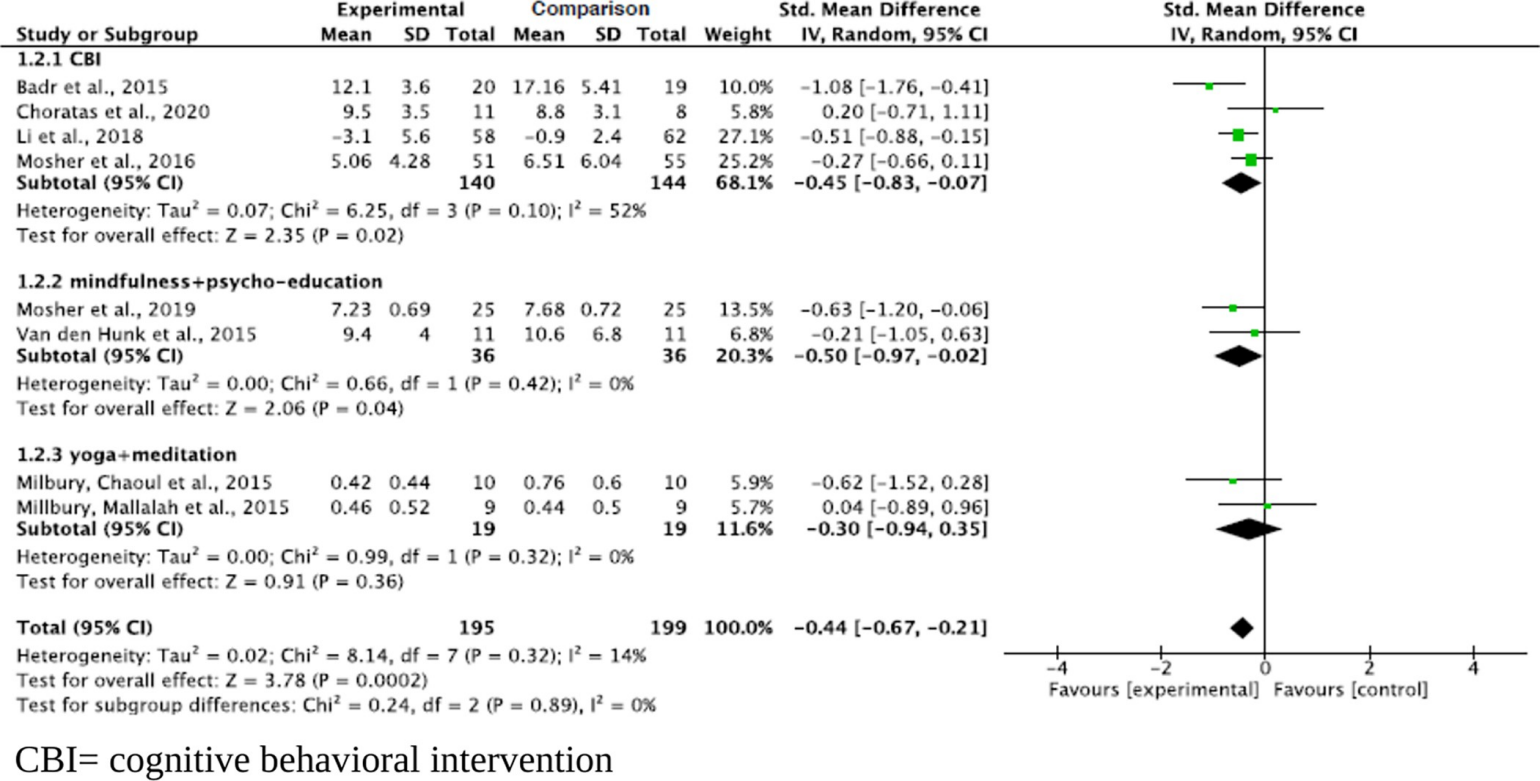
Interventions’ effect on the anxiety of informal caregivers for lung cancer patients by intervention types. CBI = cognitive behavioral intervention.

As against comparison groups, the cognitive-behavioral intervention, and the interventions combining mindfulness and psycho-education, significantly decreased the level of anxiety of informal caregivers for lung cancer patients (p = 0.02 and p = 0.04, respectively). Although a decrease in anxiety levels was also noticed on the yoga combined with meditation method, the decrease was not significant (p = 0.36). The pooled summary effect of the cognitive-behavioral intervention, and the mindfulness with psycho-education intervention, showed that caregivers in the intervention groups reduced their risk for anxiety by half as opposed to the comparison group post intervention (SMD, -0.45; 95% CI, -0.83, -0.07; and SMD, -0.50; 95% CI, -0.97, -0.02; respectively). The subgroup analysis showed a significant decrease in heterogeneity across the studies on the absent of heterogeneity of mindfulness with psycho-education intervention studies, and yoga with meditation intervention studies (Tau^2^ = 0.00, ChI^2^ = 0.66, df = 1, p = 0.42, I^2^ = 0% and Tau^2^ = 0.00, ChI^2^ = 0.99, df = 1, p = 0.32, I^2^ = 0%, respectively) (Fig 3).

#### Effect by mode of contact

**Fig 4:**
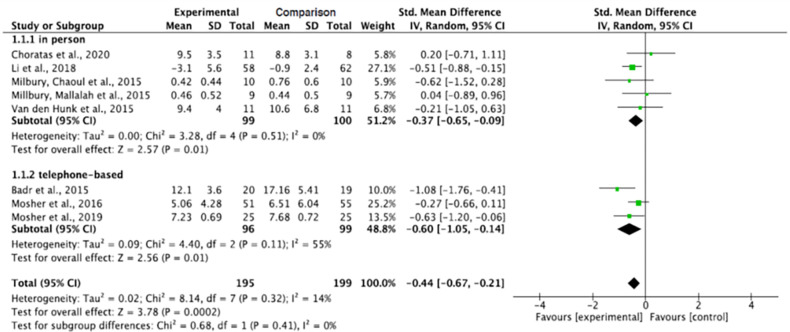
Interventions’ effect on the anxiety of informal caregivers for lung cancer patients by mode of contact.

Interventions using both in person and telephone-based contact methods significantly decreased levels of anxiety for informal caregivers (p = 0.01). The pooled summary effect of the in person and telephone-based interventions showed that caregivers in these intervention groups were 0.37 and 0.60 lower at risk for anxiety in opposition with the comparison group post intervention (SMD, -0.37; 95% CI, -0.65, -0.09; and SMD, -0.60; 95% CI, -1.05, -0.14; respectively). The subgroup analysis showed an absence of heterogeneity across the in person intervention studies (Tau^2^ = 0.00, ChI^2^ = 3.28, df = 4, p = 0.51, I^2^ = 0%), and an increase of heterogeneity across the telephone-based intervention studies (Tau^2^ = 0.09, ChI^2^ = 4.40, df = 2, p = 0.11, I^2^ = 55%) (Fig 4).

#### Effect by group versus individual delivery

**Fig 5:**
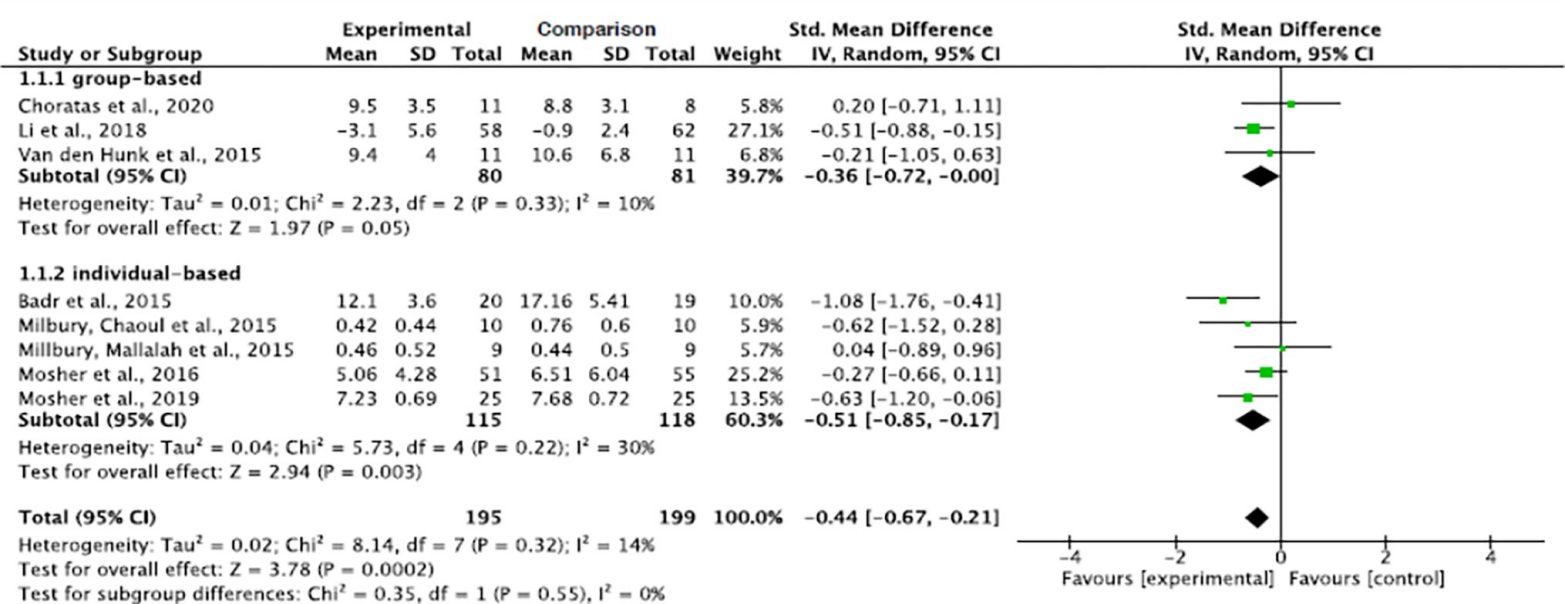
Interventions’ effect on the anxiety of informal caregivers for lung cancer patients by group versus individual delivery.

Individual-based interventions demonstrated significant decreasing levels of anxiety in informal caregivers (p = 0.003). Although a decreased anxiety level was also noticed in the group-based intervention method, the decrease narrowly failed to reach significance (p = 0.05). The pooled summary effect of the individual-based interventions showed that caregivers in the intervention group were about 0.51 lower at risk for anxiety than the comparison group post intervention (SMD, -0.51; 95% CI, -0.85, -0.17). The subgroup analysis showed a low heterogeneity across the group-based intervention studies (Tau^2^ = 0.01, ChI^2^ = 2.23, df = 2, p = 0.33, I^2^ = 10%) and individual based intervention studies (Tau^2^ = 0.04, ChI^2^ = 5.73, df = 4, p = 0.22, I^2^ = 30%) (Fig 5).

### Effect on the depression of informal caregivers for lung cancer patients

#### Total effect

**Fig 6:**
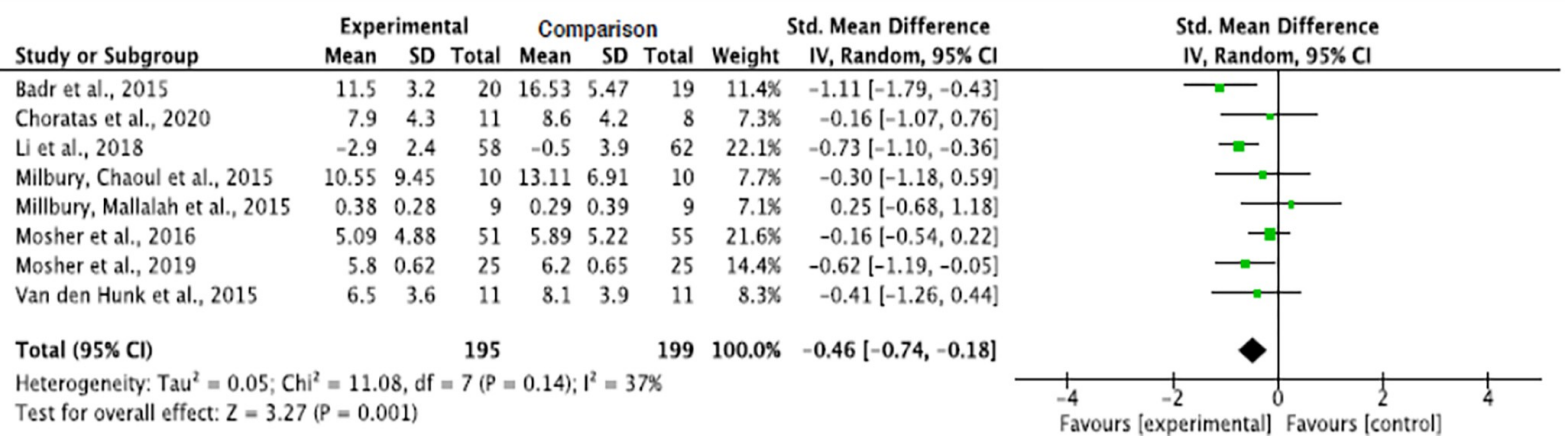
Interventions’ effect on the depression of informal caregivers for lung cancer patients.

Interventions for informal caregivers of lung cancer patients significantly decreased the caregivers’ depression levels as opposed to the comparison groups (p = 0.001). The pooled summary effect of the included interventions showed that caregivers in the intervention group were 0.46 lower at risk for depression than the comparison group post intervention (SMD, -0.46; 95% CI, -0.74, -0.18). A low heterogeneity was noticed across the study results (Tau^2^ = 0.05, ChI^2^ = 11.08, df = 7, p = 0.14, I^2^ = 37%) (Fig 6).

#### Effect by intervention types

**Fig 7:**
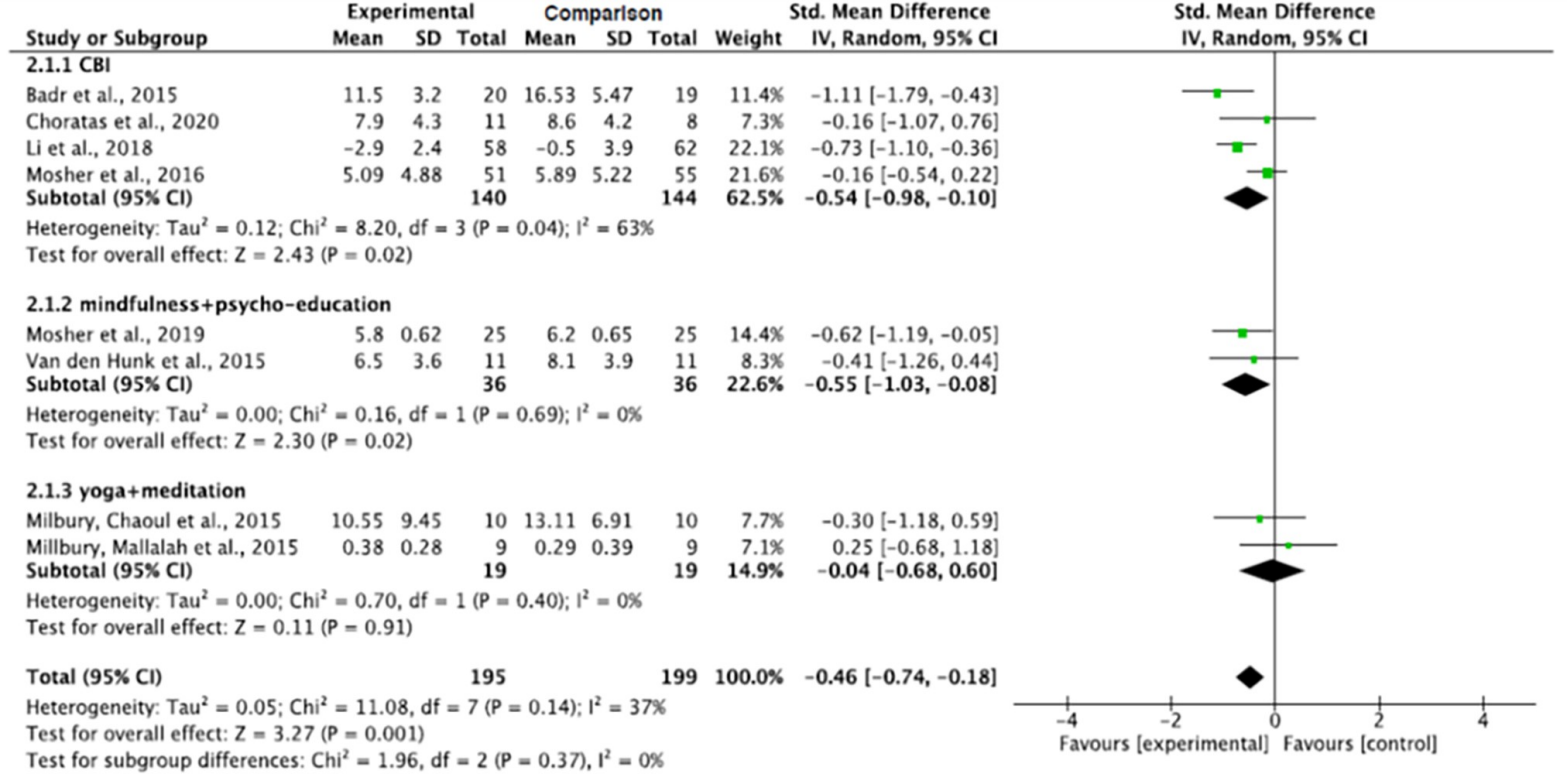
Interventions’ effect on the depression of informal caregivers for lung cancer patients by intervention types.

The cognitive-behavioral intervention, and the mindfulness with psycho-education interventions, significantly decreased depression in informal caregivers of lung cancer patients (p = 0.02). Although a decreased depression level was also noticed with the yoga combined with meditation method, the decrease was not significant (p = 0.91). The pooled summary effect of the cognitive-behavioral intervention, and the mindfulness with psycho-education interventions, showed that caregivers in the intervention groups were about 0.54 and 0.55 lower at risk for depression as opposed to the comparison group post intervention (SMD, -0.54; 95% CI, -0.98, -0.10; and SMD, -0.55; 95% CI, -1.03, -0.08; respectively). The subgroup analysis showed a significant decrease in heterogeneity across the studies and the absence of heterogeneity of mindfulness combined with psycho-education, and yoga combined with meditation intervention studies (Tau^2^ = 0.00, ChI^2^ = 0.16, df = 1, p = 0.69, I^2^ = 0% and Tau^2^ = 0.00, ChI^2^ = 0.70, df = 1, p = 0.40, I^2^ = 0%, respectively) (Fig 7).

#### Effect by mode of contact

**Fig 8:**
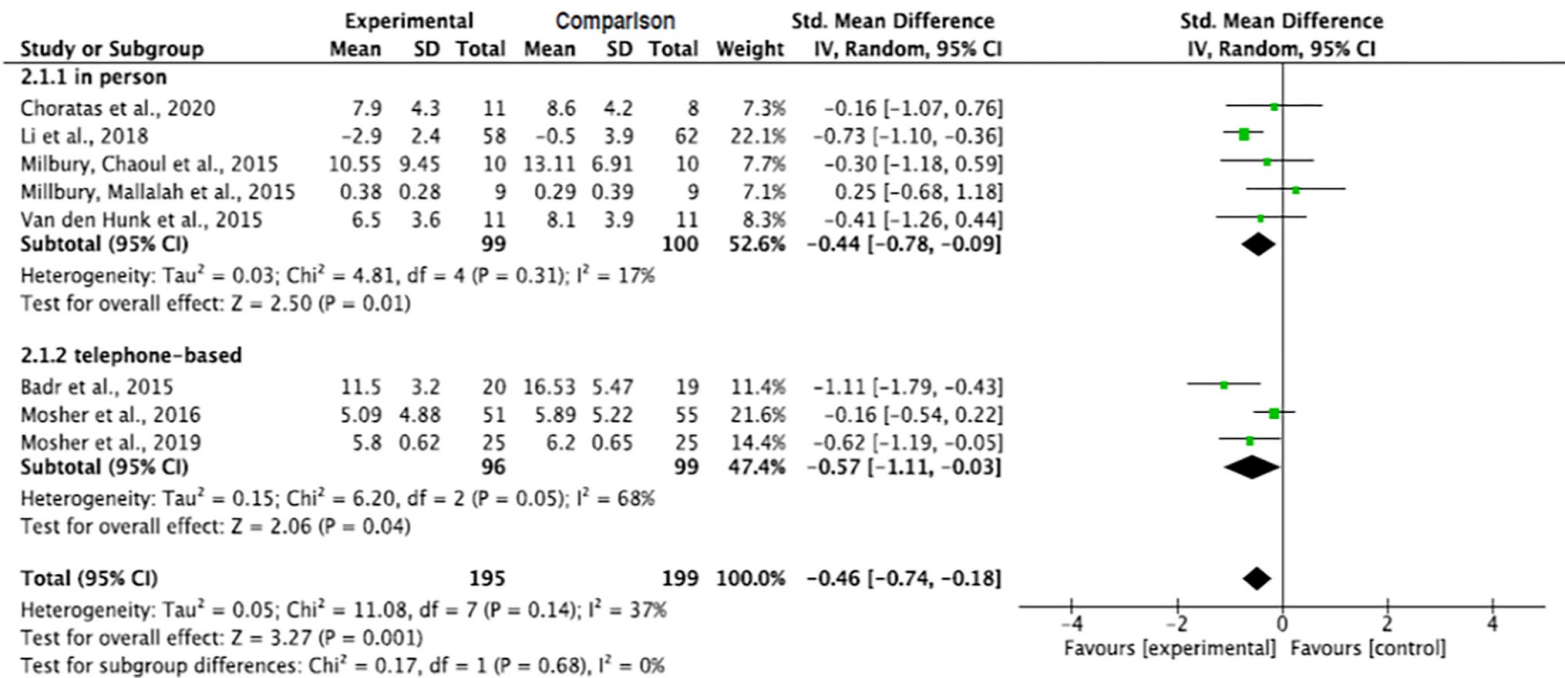
Interventions’ effect on the depression of informal caregivers for lung cancer patients by mode of contact.

Both in-person and telephone-based interventions significantly decreased the level of depression in informal caregivers of patients with lung cancer (p = 0.01 and p = 0.04, respectively). The pooled summary effect of the in person and telephone-based interventions showed that caregivers in the intervention groups were 0.44 and 0.57 lower at risk for depression as opposed to the comparison group post intervention (SMD, -0.44; 95% CI, -0.78, -0.09; and SMD, -0.57; 95% CI, -1.11, -0.03, respectively). The subgroup analysis showed decreased heterogeneity across in-person intervention studies (Tau^2^ = 0.03, ChI^2^ = 4.81, df = 4, p = 0.31, I^2^ = 17%) but increased heterogeneity across the telephone-based intervention studies (Tau^2^ = 0.15, ChI^2^ = 6.20, df = 2, p = 0.05, I^2^ = 68%) (Fig 8).

#### Effect by group versus individual delivery

**Fig 9:**
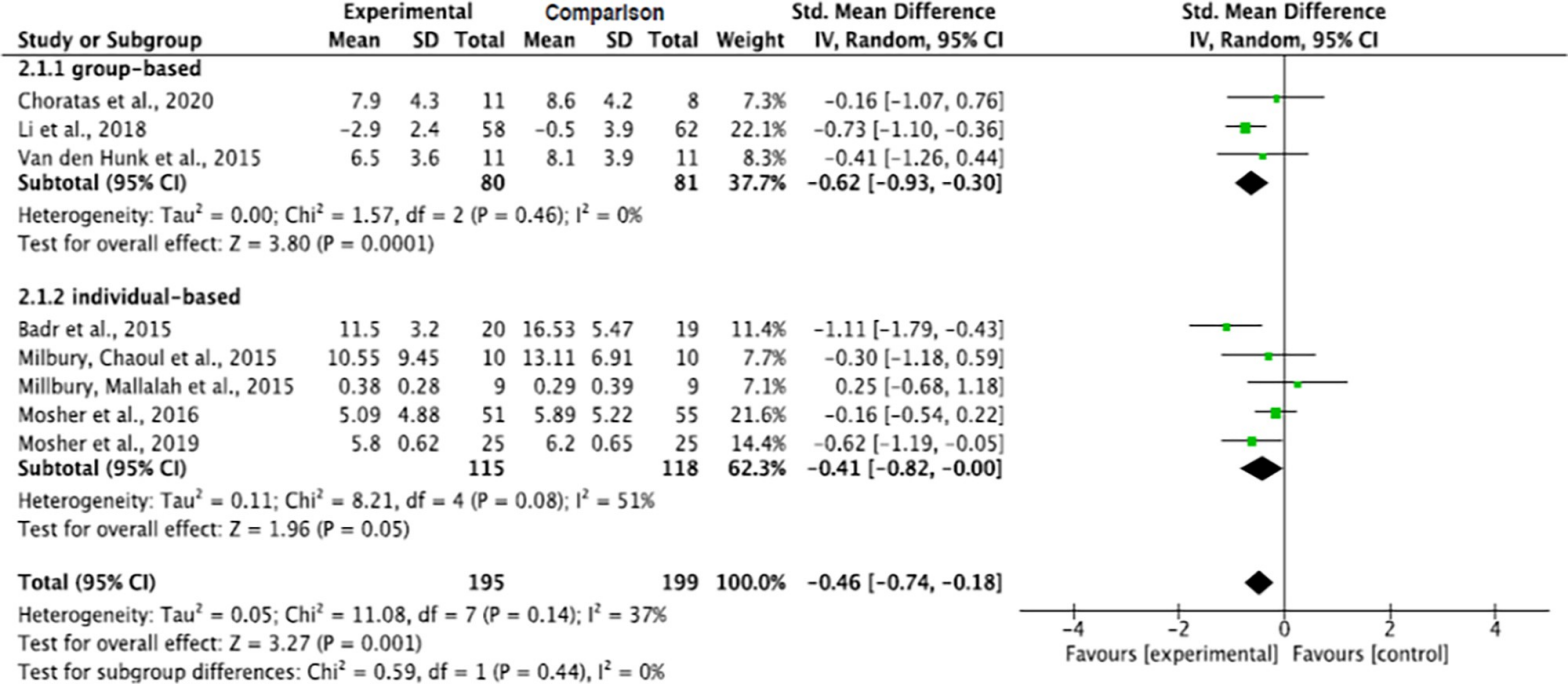
Interventions’ effect on the depression of informal caregivers for lung cancer patients by group versus individual delivery.

Results showed that when contrasted with the comparison groups, the group-based interventions significantly decreased depression in informal caregivers for lung cancer patients (p = 0.0001). Although a decreased depression level was also noticed in the individual-based intervention method, the decrease narrowly failed to reach significance (p = 0.05). The pooled summary effect of the group-based interventions showed that caregivers in the intervention group were about 0.62 lower at risk for depression than the comparison group post intervention (SMD, -0.62; 95% CI, -0.93, -0.30). The subgroup analysis showed decreased heterogeneity across the group-based intervention studies (Tau^2^ = 0.00, ChI^2^ = 1.57, df = 2, p = 0.46, I^2^ = 0%) but increased across the individual-based intervention studies (Tau^2^ = 0.11, ChI^2^ = 8.21, df = 4, p = 0.08, I^2^ = 51%) (Fig 9).

### Publication bias

**Fig 10:**
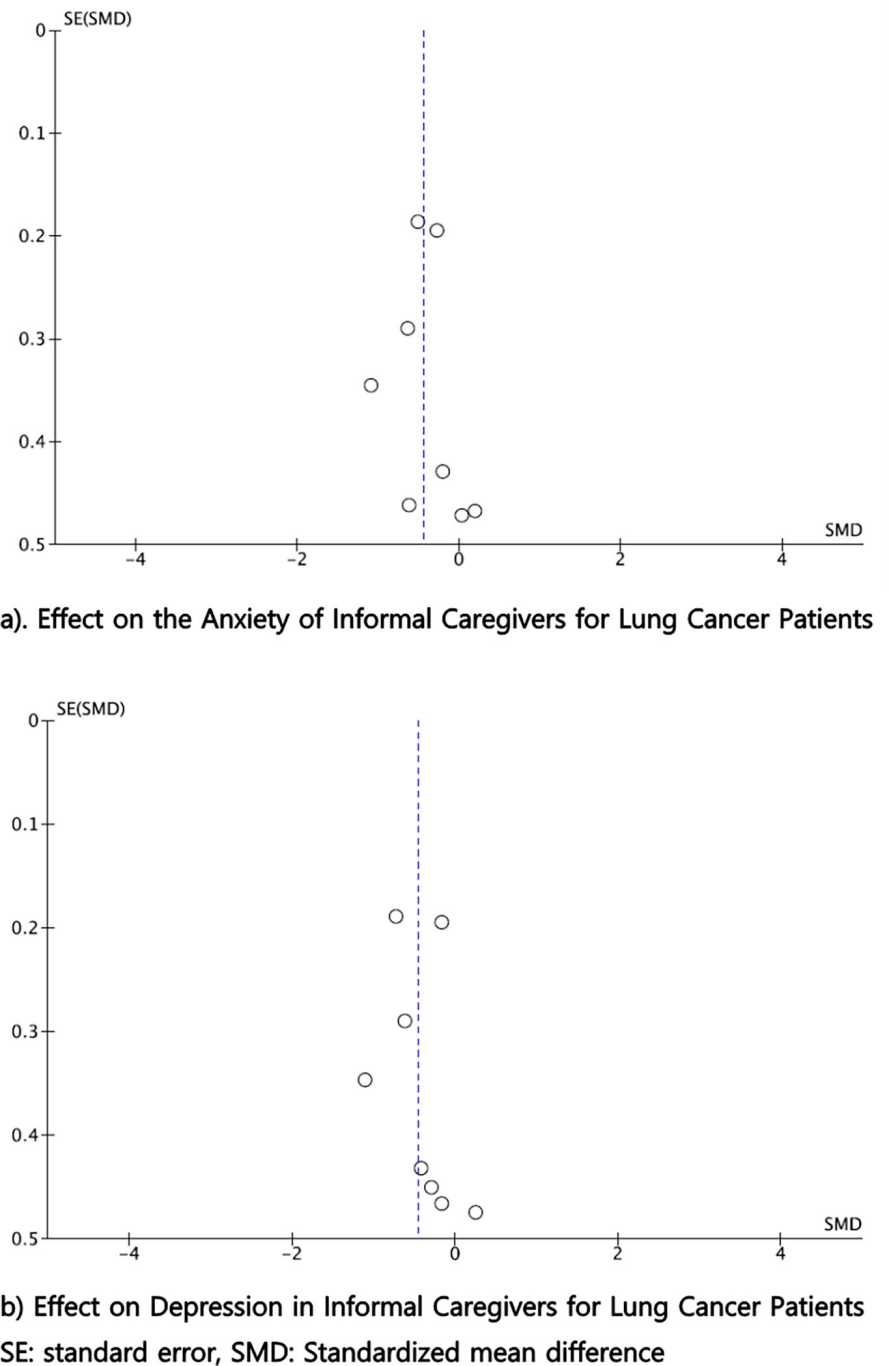
Funnel plots. **a.** Effect on the Anxiety of Informal Caregivers for Lung Cancer Patients. SE: standard error, SMD: Standardized mean difference. **b.** Effect on Depression in Informal Caregivers for Lung Cancer Patients. SE: standard error, SMD: Standardized mean difference.

Respective funnel plots were generated for each main outcome of interest to evaluate publication bias. The distribution of data points provided limited evidence for small study publication bias (Fig 10A and 10B).

## Discussion

This systematic review and meta-analysis found that non-pharmacological interventions have generally been successful in significantly decreasing anxiety and depression on caregivers of people with lung cancer. Subgroup analyses revealed that relative effectiveness on both anxiety and depression may depend on specific intervention types, mode of contact, and group versus individual delivery.

First, regarding intervention types, there have been few systematic reviews and meta-analyses on non-pharmacological interventions on anxiety and depression in informal caregivers of patient populations other than lung cancer. The findings of this review are consistent with a previous systematic review and meta-analysis, which has found significant effects on meditation intervention on informal caregivers of chronic illness patients [20]. After 27 randomized controlled trials evaluating the association of meditation interventions at an average of eight weeks following intervention initiation in informal caregivers of chronic illness patients, a meta-analysis reported that meditation interventions were associated with significant improvement in anxiety (effect size 0.53, 95% CI 0.06 to 0.99) and in depression (effect size 0.49, 95% CI 0.24 to 0.75) [20]. Interestingly, previous meta-analyses papers reported little significant effects of cognitive behavioral therapies on both anxiety and depression in informal caregivers of patients with cancer of various kinds beyond just lung cancer, including: informal caregivers of cancer patients and cancer survivors [21], psychosocial interventions with informal caregivers of cancer patients [22], and insignificant effects of psychosocial interventions on anxiety in caregivers of advanced cancer patients [23]. Cognitive behavioral therapy refers to intervention strategies such as cognitive restructuring, coping skills training or stress and anxiety management [36]. A meta-analysis [21] analyzed 36 studies using cognitive behavioral therapy and found a small statistically significant effect of cognitive behavioral therapy on the common psychological complaints of cancer patients’ caregivers, such as anxiety and depression (Hedge’s *g* = 0.08, *p* = 0.014). This is inconsistent with our findings in this review that cognitive behavioral interventions have small to large effects on reducing anxiety and depression in informal caregivers of lung cancer patients. Furthermore, the present findings are inconsistent with a meta-analysis that revealed significant small to moderate effects on both anxiety and depression in yoga interventions for cancer patients [37]. More specifically, a systematic review and meta-analysis of 26 yoga interventions for cancer patients, including a majority of breast cancer patients, revealed evidence of significant small to medium effects of yoga on depression (g = -0.419, 95% CI -0.558 to -0.281) and anxiety (g = -0.347, 95% CI -0.473 to -0.221) [37]. Different study characteristics such as cancer patients vs. caregivers, or cancer vs. lung cancer may result in different study results (e.g., intervention effectiveness).

Second, for mode of contact and group versus individual delivery, findings of this review, that in person and telephone-based interventions, and individual- and group-based interventions decreased anxiety and depression in informal caregivers of patients with lung cancer, are similar to findings from previous such meta-analyses. For example, a meta-analysis of 29 randomized trial studies with family caregivers of cancer patients reported that both face to face intervention (Hedges’ g 1.06, 95% CI 0.42 to 1.71, p<0.05) and group-based intervention (Hedges’ g 1.01, 95% CI 0.39 to 1.63, p<0.001) significantly improved coping strategies [38]. Although this review included individual- and telephone-based interventions for informal caregivers, internet-based interventions on informal caregivers’ mental health were previously investigated as effective delivery methods. For example, a meta-analysis study [39] examined the impact of internet-based interventions on informal caregiver mental health outcomes. It revealed the beneficial effects of internet-based intervention programs by decreasing a mean of 0.40 (95% CI -0.58 to -0.22) for anxiety among informal caregivers of adults with cancer, dementia, or stroke [39]. Furthermore, information platforms (e.g., smartphone applications) are considered useful in providing information in a timely manner [8]. Thus, future research needs to explore various and innovative intervention delivery methods for the psychological health of informal caregivers of lung cancer patients.

### Strengths and limitations

Despite the fact that the well-being of lung cancer patients’ informal caregivers is crucial, a few evidence-based interventions addressing anxiety and depression in such caregivers are available at present. The strengths of this systematic review and meta-analysis study are: a) providing the first evidence summary on the effectiveness of non-pharmacological interventions on anxiety and depression for lung cancer patients’ informal caregivers, b) including newly published intervention studies on these issues, and c) performing new subgroup analyses compared to previous reviews.

This systematic review and meta-analysis had limitations that we should be aware of. Firstly, only eight studies were included in this review and two or four studies were analyzed in subgroup analysis. We found significant decreases in anxiety and depression, though only two to four studies in each subgroup were included, which may limit the strength of our results. While some indicate that at least two studies are needed to conduct a meta-analysis [40], others suggest that meta-analyses with very small numbers of studies may underestimate heterogeneity [41]. Because our subgroups in this meta-analysis are relatively small, heterogeneity results should be interpreted with caution. Lastly, we included studies published only in English, which may reduce the generalizability of our results to non-English speaking countries.

## Conclusions

Psychological problems such as anxiety and depression in informal caregivers of patients with lung cancer are closely interrelated with the health outcomes of the patients [4–7]. We should attempt to improve positive health outcomes in both populations. The findings of this meta-analysis show potential beneficial effects of cognitive behavioral and mindfulness-based, telephone-based, individual- or group-based interventions on anxiety and depression in informal caregivers of patients with lung cancer. Further research is needed to develop and test in order to find the most effective intervention contents and delivery methods by comparing different psychological interventions head-to-head in trials.

## Supporting information

S1 Checklist PRISMA 2020 checklist. (DOCX)

S1 Appendix (DOCX)
