# Supplementary material for: Non-pharmacological interventions on anxiety and depression in lung cancer patients’ informal caregivers: A systematic review and meta-analysis
Source: PLoS One. 2023 Mar 13;18(3):e0282887. doi: 10.1371/journal.pone.0282887 (PMC10010534; doi:10.1371/journal.pone.0282887)
Supplement: S1 Appendix — (DOCX) [file pone.0282887.s002.docx]

APPENDIX

PubMed Search Strategy:

(caregiver[MeSH Terms] OR caregivers[Mesh Terms] OR caregivers OR family OR relatives OR carer) AND (lung cancer[MeSH Terms] OR lung neoplasm[MeSH Terms] OR lung cancer OR lung neoplasm OR lung tumor) AND (cognitive psychotherapy [MeSH Terms] OR psychotherapy OR social support [MeSH Terms] OR social support OR patient education [MeSH Terms] OR educational intervention OR education training OR patient education OR interdisciplinary communication [MeSH Terms] OR interdisciplinary communication) AND (anxiety [MeSH Terms] OR anxiety disorders [MeSH Terms] OR generalized anxiety disorder OR depression [MeSH Terms] OR depressive disorder [MeSH Terms] OR depressive symptoms OR major depressive disorder)
